# Evaluation of Dysplasia Grading in Colorectal Adenomas According to Current Guidelines: A Nationwide Survey Among Pathologists in Türkiye*

**DOI:** 10.5146/tjpath.2026.12897

**Published:** 2026-05-30

**Authors:** Eylul Gun, Betul Bolat Kucukzeybek, David Driman, Aysegul Akder Sari

**Affiliations:** Department of Cellular Pathology, Basildon & Thurrock University Hospital, Mid and South Essex NHS Foundation Trust, BASILDON, ESSEX, UNITED KINGDOM; Department of Pathology, Izmir Katip Celebi University Ataturk Training and Research Hospital, İzmİr, TÜRKİYE; Department of Pathology and Laboratory Medicine, London Health Sciences Centre and Western University, London, Ontario, Canada

**Keywords:** Survey, Grading, Dysplasia, Colorectal adenoma

## Abstract

Objective: Due to its effect on polypectomy surveillance, dysplasia grading is essential in colorectal adenomas. We aimed to investigate the terminologies used for high-grade dysplasia in routine practice if any, and to assess the practice patterns of dysplasia grading among Turkish pathologists with reference to current guidelines.

Material and Methods: A survey including seven images of colorectal adenomas with low-grade and high-grade dysplasia was sent to pathologists in Türkiye. The images were selected from the Canadian National Polyp Guidelines. The pathologists were asked to grade the dysplasia as in their routine practice.

Results: A total of 324 pathologists (including 48 senior residents and 176 gastrointestinal pathologists) participated in the survey. Among staff pathologists, the rate of agreement of dysplasia according to the guidelines was as follows; for colorectal adenomas with low-grade dysplasia, Image 1 (with low-grade cytology)-97.1%, Image 2 (with focal cribriformity)-6.2%, Image 5 (with high-grade cytology)-26.8%, and Image 6 (with slightly high-grade cytology)-48.9%. For colorectal adenomas with high-grade dysplasia, the agreement ratio was >91% (range 91.6-99.6%). However, intramucosal adenocarcinoma was the preferred terminology by 43.8%, 68.5%, and 50% of the participants, respectively. The results were similar for residents.

Conclusion: Among pathologists in Türkiye, in contrast to current guidelines for colorectal adenomas, 1. Intramucosal adenocarcinoma is still a commonly used terminology, 2. High-grade cytological features are over-relied upon, 3. Small foci of architectural abnormality are overcalled. Therefore, strategies to increase the usage of established practice guidelines should be developed. A national polyp guideline seems to be necessary for an attempt to standardize the reporting of colorectal adenomas.

## INTRODUCTION

Colorectal carcinomas constitute the third most common cause of cancer deaths in both males and females in the United States, according to Cancer Statistics data (1). Global Cancer Statistics reports that colorectal cancer was the third most commonly diagnosed and second leading cause of cancer death worldwide in 2020 (2). Given the significant impact on morbidity and mortality, population-based colorectal cancer screening programs have been instituted. As a result of these programs, colorectal adenomas (CRA) with the potential to progress to colorectal cancers are among the most frequently encountered cases in pathology practice.

Accurate grading of dysplasia is important in CRAs as it affects polypectomy surveillance (3,4). There are interobserver differences among pathologists in the grading of dysplasia of CRAs. Since high-grade dysplasia (HGD) in colorectal polyps falls under the category of “advanced adenoma,” they require more frequent follow-up (5). To avoid overtreatment (e.g. surgical resection), terms such as intramucosal carcinoma are discouraged in CRAs, as these lesions are considered to have minimal/no risk of metastatic spread. Current guidelines, including the World Health Organization (WHO) 2019, classify adenomatous dysplasia into low-grade and high-grade, without defining intramucosal carcinoma or carcinoma in situ as distinct categories; therefore, high-grade dysplasia encompasses both of these terms (6). In order to standardize the grading system, guidelines describing the cytological and architectural features of CRAs have been developed (5,7,8). In addition, studies have been conducted to contribute to the surveillance after colonoscopic polypectomy (8–10).

The lack of standardized guidelines in our country may cause deficiencies in the standardization of pathology reports of CRAs and surveillance follow-up. Therefore, in this study, we aimed to assess the practice patterns of dysplasia grading among Turkish pathologists with reference to current guidelines. Our goals were to 1) assess the participants’ understanding/knowledge of the current grading rules, 2) determine whether other terminologies such as intramucosal adenocarcinoma are used in place of high-grade dysplasia, which is discouraged in current guidelines, and 3) identify the impact of associated factors such as gastrointestinal pathology experience and years of specialization on grading practices.”

## MATERIAL and METHODS

**Figure 1:**
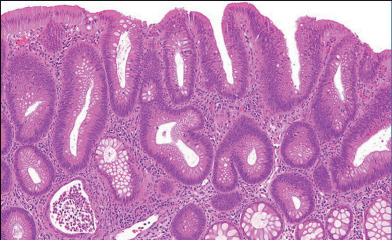
Low-grade dysplasia with no architectural complexity and low-grade cytological features

**Figure 2:**
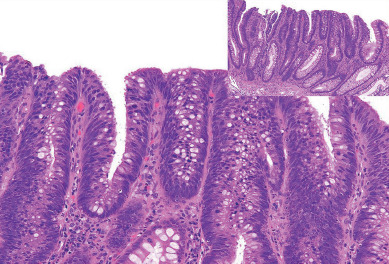
Low-grade dysplasia with no architectural complexity and slightly high-grade cytological features

**Figure 3:**
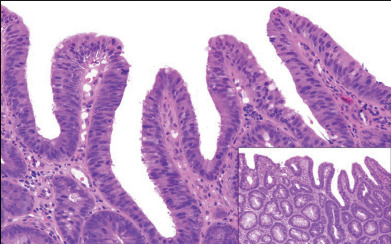
Low-grade dysplasia with no architectural complexity and high-grade cytological features

**Figure 4:**
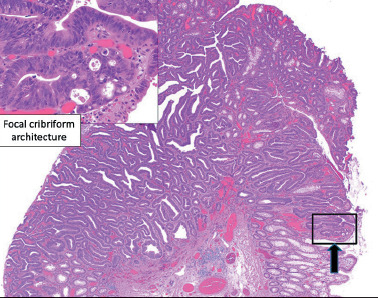
Low-grade dysplasia with focal cribriform architecture (inset) and high-grade cytological features

**Figure 5:**
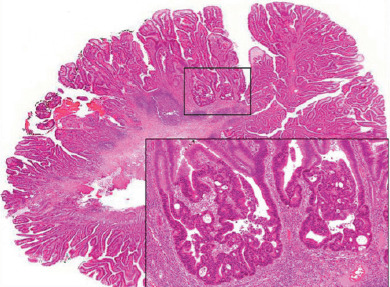
High-grade dysplasia with cribriformity and high-grade cytologic features

**Figure 6:**
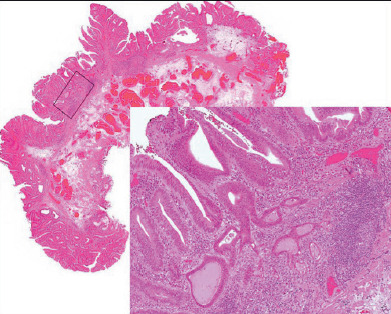
High-grade dysplasia with lamina propria invasion

**Figure 7:**
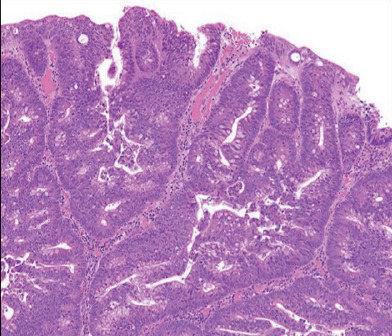
High-grade dysplasia with architectural complexity with cribriform formations and high-grade cytologic features

A survey including seven images of CRAs with LGD and HGD was sent to pathologists in Türkiye via e-mail and social media channels. The images were selected from the Canadian National Polyp Guidelines; these clearly reflect the degree of dysplasia, were chosen by consensus by the authors, and were regarded as reliable and demonstrative. The images were not sent in order. Cases with LGD were represented by questionnaire images 1, 2, 5, and 6 (Figure 1,Figure 2,Figure 3,Figure 4), and cases with HGD were represented by questionnaire images 3, 4, and 7 (Figure 5,Figure 6,Figure 7). The pathologists were asked to grade the dysplasia as in their routine practice. For this purpose, multiple choices not only included LGD and HGD but also adenocarcinoma in-situ and intramucosal adenocarcinoma.

## Statistical Analysis

The survey results were analyzed using “SPSS 24.0 for Windows” (IBM, Chicago, IL, USA). All descriptive data were presented as frequencies and percentages. Statistical analyses were conducted using the chi-square test and Fisher’s exact test where appropriate. Interrater variability among numerous raters was assessed using Fleiss’ Kappa (11). A p-value of less than 0.05 was considered statistically significant.

Besides descriptive statistics, the categorical data were compared using the chi-square test. A p-value of <0.05 was considered statistically significant.

The ethics committee approval for the study was obtained from the Non-Invasive Research Ethics Committee of Izmir Katip Celebi University (Approval number: 2021-GOKAE-0077).

## RESULTS

A total of 324 pathologists consisting of 48 senior residents and 276 staff pathologists, participated in the survey. Among the staff pathologists, 176 (63.8%) had a specific interest and experience in gastrointestinal (GI) pathology. Most of the participants had more than five years of experience. The places of work varied and were grouped under two categories: educational (university hospitals and governmental education-training hospitals/affiliated university hospitals) and non-educational (government hospitals and private hospitals/laboratories). The years of specialization category was grouped as <10 years and >10 years. No statistically significant differences were found in the agreement ratios based on the places of work, years of specialization, and interest and specialized experience in GI pathology (p>0.05). The sole statistically significant distinction observed between GI-experienced and non-GI pathologists pertained to Image 5 Figure 4, revealing that experienced GI pathologists exhibit a slightly diminished consideration of high-grade cytological features in the absence of architectural complexity compared to their non-GI counterparts (p=0.034).

Among staff pathologists, for all seven cases (LGD cases (Figure 1,Figure 2,Figure 3,Figure 4) and HGD cases (Figure 5,Figure 6,Figure 7), the Fleiss Kappa value was calculated as 0.66 indicating substantial agreement among pathologists. The 95% confidence interval for the Fleiss kappa value was 0.62 to 0.70.

The agreement rates for dysplasia in LGD cases, according to the guidelines, were as follows: For CRAs with LGD (a total of four cases): Image 1 with no architectural complexity and low-grade cytological features – 97.1% (Figure 1), Image 6 with no architectural complexity and slightly high-grade cytological features – 48.9% (Figure 2), Image 5 with no architectural complexity and high-grade cytological features - 26.8% (Figure 3), and Image 2 with focal cribriformity and high-grade cytological features – 6.2% (Figure 4). The results are listed in Table 1 and were similar for residents.

**Table 1:** ** **Answers to each image in the survey by the participant pathologists.

**Guideline Diagnosis** **(Histological features)**	**Pathologists answering as LGD % (n)**	Pathologists answering as *HGD % (n)
Image 1- LGD (Architectural complexity Ø Cytology– Low-grade)	97.1 (268)	2.9 (8)
Image 2- LGD (Architectural - Focal cribriformity, Cytology– High-grade)	6.2 (17)	93.8 (259)
Image 3 - HGD (Architectural - More than focal cribriformity)	0.4 (1)	99.6 (275)
Image 4 - HGD (Infiltrative glands in the lamina propria)	8.3 (23)	91.6 (253)
Image 5 - LGD (Architectural complexity Ø Cytology– High-grade)	26.8 (74)	73.2 (202)
Image 6 - LGD (Architectural complexity Ø Cytology– Slightly high-grade)	48.9 (135)	51.1 (141)
Image 7 - HGD (Architectural - Diffuse cribriform glands)	4.3 (12)	95.6 (264)

**LGD:** Low-grade dysplasia, **HGD:** High-grade dysplasia, *HGD category in this table encompasses all responses including HGD, in situ adenocarcinoma and intramucosal adenocarcinoma

Different terminologies were used among researchers for CRAs with HGD (Images 3, 4, and 7 – Figure 5,Figure 6,Figure 7), such as intramucosal carcinoma and in-situ adenocarcinoma. The response rates of participants for each group of images are available in Table 2.

**Table 2:** The response rates of participants for each group of images.

**Guideline Diagnosis** **(Histological features)**	**LGD % (n)** **1) GI vs non-GI** **2) Years of education (<10 years vs >10 years)** **3) Place of work (educational vs non-educational)**	**Other terminologies for HGD % (n)** **1) GI vs non-GI** **2) Years of education (<10 years vs >10 years)** **3) Place of work (educational vs non-educational)**
Image 1- LGD (Architectural complexity Ø Cytological features – Low-grade)	1) 97.7% (172/176) vs 96% (96/100) (p=0.466)* 2) 95.8% (159/166) vs 99% (109/110) (p=0.15)* 3) 98.8%(159/161) vs 94.8%(109/115) (p=0.71)*	
Image 2- LGD (Architectural - Focal cribriformity Cytological features – High-grade)	1) 6.8% (12/176) vs 5% (5/100) (p=0.546) 2) 6.6% (11/166) vs 5.5% (6/110) (p=0.692) 3) 7.5% (12/161) vs 4 % (5/115) (p=0.290)	
Image 3 - HGD (Architectural - More than focal cribriformity)		**In-situ adenoca: 21% (58) / ImA: 43.8% (121)** 1) 17.6% (31) vs 27% (27) / 45.5% (80) vs 41% (41) 2) 19.9% (33) vs 22.7% (25) / 47.6% (79) vs 38.2% (42) 3) 19.3% (31) vs 23.5% (27) / 46.6% (75) vs 40% (46)
Image 4 - HGD (Infiltrative glands in the lamina propria)		**In-situ adenoca: 5.4% (15) / ImA: 68.5% (189)** 1) 5.7% (10) vs 5% (5) / 70.5% (124) vs 65% (65) 2) 4.8% (8) vs 6.4% (7) / 70.5% (117) vs 65.5% (72) 3) 3.7% (6) vs 7.8% (9) / 69.6% (112) vs 67% (77)
Image 5 - LGD (Architectural complexity Ø Cytological features – High-grade)	1) 31% (55/176) vs 19% (19/100) **(p=0.027)** 2) 30% (50/166) vs 21.8% (24/110) (p=0.127) 3) 26% (42/161) vs 27.8% (32/115) (p=0.748)	
Image 6 - LGD (Architectural complexity Ø Cytological features – Slightly high-grade)	1) 49% (87/176) vs 48% (48/100) (p=0.819) 2) 51% (85/166) vs 45.5% (50/110) (p=0.349) 3) 50% (81/161) vs 47% (54/115) (p=0.583)	
Image 7 - HGD (Architectural - Diffuse cribriform glands)		**In-situ adenoca: 16.3% (45) / ImA: 50% (138)** 1) 17.6% (31) vs 14% (14) / 48.9% (86) vs 52% (52) 2) 12.7% (21) vs 21.8% (24) / 55.4% (92) vs 41.8% (46) 3) 19.3% (31) vs 12.2% (14) / 49.7% (80) vs 50.4% (58)

*p values were calculated using the Fischer exact test. **LGD: **Low-grade dysplasia, **HGD: **High-grade dysplasia, **GI:** Gastrointestinal, **Adenoca: **Adenocarcinoma, **ImA:** Intramucosal adenocarcinoma

**Figure 8:**
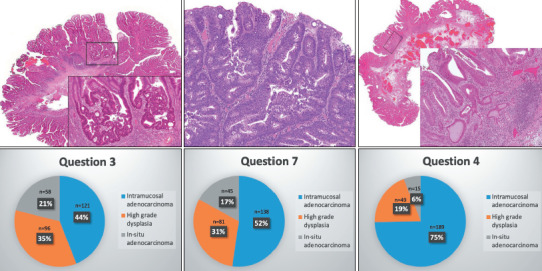
The distribution of different terminology usage in cases with high-grade dysplasia according to the guidelines

When all these terminologies were grouped under the title of HGD, the agreement ratio with the guideline was >91% (range 91.6-99.6%). However, intramucosal adenocarcinoma was the preferred terminology by 44%, 74.7%, and 52.3% of the participants, respectively, and in-situ adenocarcinoma was used by 21.1%, 5.9%, and 17%. The distribution of different terminology usage in cases with HGD according to the guidelines is shown in Figure 8 and Table 2.

The use of “intramucosal adenocarcinoma” terminology increased in cases with lamina propria invasion (Figure 6) and as the glands exhibiting cribriformity increased (Figure 7). The results were similar for residents. Regarding the use of the term “intramucosal adenocarcinoma”, no statistical difference was observed between pathologists who were interested in GI and those who were not (p>0.05).

Among the staff pathologists, the WHO Classification of Tumours of the Digestive System (WHO-GI book) was the most preferred reference when reporting dysplasia, followed by GI pathology books; the rest referred to various other sources. (The ratios were 64.3%, 21%, and 15% for GI-interested staff versus 52%, 26%, and 22% for non-GI-interested staff, respectively. For residents, these percentages were 79%, 18%, and 2%, respectively.) Most of the participants (92.3%) said “Yes” to the question asking about the need for a national guideline for grading CRAs (Question 9: To standardize the reporting of colorectal adenomas, do you think it would be useful to develop a national guideline that describes the structural and cytological features and includes photographs and examples or to present a similar guide as an example?).

## DISCUSSION

The current guidelines for grading dysplasia in colorectal adenomas, such as the 2019 WHO Classification, the Royal College of Pathologists, the Royal College of Pathologists of Australasia, and the International Collaboration on Cancer Reporting (ICCR) guidelines, as well as the Canadian polyp guidelines, all point out a two-tiered stratification system (low-grade and high-grade). Use of the terms mild, moderate, or severe dysplasia is discouraged. Furthermore, the term HGD is preferred over terms such as carcinoma in situ and intraepithelial carcinoma (6,12–15). There are no consensus guidelines or suggestions to follow current guidelines for grading CRAs in our country. Our study showed that although 60% of the pathologists who participated in the survey stated that they use the 2019 WHO Classification as a reference, there was variability and inconsistencies in the preferred terminologies.

When assessing the degree of dysplasia in CRAs, diagnosis is mainly based on architectural features, supported by appropriate cytological features. A conventional adenoma has at least low-grade dysplasia by definition (16). The architectural features of HGD include prominent complex glandular crowding, glandular irregularity, cribriform architecture, and intraluminal necrosis. These changes can often be seen at low power. It should be noted that glandular crowding alone is not enough to grade as HGD; the architectural features must be accompanied by cytological features, which include loss of polarity, irregular nuclear stratification through the entire thickness of the epithelium, significantly enlarged nuclei with dispersed chromatin, prominent nucleoli, atypical mitotic figures, and prominent apoptosis. These changes must be present in at least two crypts for an HGD diagnosis.

Careful consideration of both architectural and cytological features should be performed when assessing the presence of HGD. Caution should be taken not to over-diagnose HGD. Some problems that pathologists face while grading dysplasia include overreliance on cytological abnormalities, overestimating the architectural complexity, and overcalling surface changes (6,12–15). Our survey results were also consistent with these findings. The agreement ratio for LGD was 97% when the adenoma had low-grade cytological features and no architectural complexity. However, when there were slightly high and high-grade cytological features with no architectural complexity, the agreement rate decreased to 48.9% and 26.8%, respectively. This finding highlights an overreliance on cytological abnormalities. In the case where focal cribriformity accompanied high-grade cytological features, the agreement rate was even lower, with only 6.2% of the pathologists grading it as LGD, which shows there is a high rate of overestimating focal architectural complexity among pathologists. The term “intramucosal adenocarcinoma,” on the other hand, has caused discrepancies between the East and the West. In countries with no published guidelines like ours, this discrepancy also creates high levels of interobserver disagreement in the grading of dysplasia. This has been shown in different studies worldwide (8,10,17). In our study, we have shown that different terminologies were used among pathologists for CRAs with HGD, such as intramucosal carcinoma and in-situ adenocarcinoma. When all these terminologies were grouped under the title of HGD, the agreement ratio with the guidelines was >91%. (range 91.6-99.6%). However, the use of intramucosal adenocarcinoma terminology increased in the presence of lamina propria invasion and when the number of crypts showing cribriformity increased. In our opinion, there might be several reasons for still using the intramucosal adenocarcinoma terminology in CRAs: “1) Some pathologists might not be aware of the current grading system. 2) Even if they are aware, they might be unwilling to use it or may not feel confident in doing so.” (As large cribriform glands in the lamina propria are characteristic of intramucosal adenocarcinoma in other parts of the GI tract.). 3) There might be a struggle in interpreting the criteria correctly. Our assumptions appeared to be supported by the responses to the ninth question in the questionnaire, where the vast majority (92.3%) of participants expressed the necessity for national colorectal polyp guidelines that encompass comprehensive cytological and architectural changes, coupled with corresponding images.

The WHO 2019 and the other guidelines that follow it recommend the use of the term HGD in the presence of lamina propria invasion with or without the invasion of muscularis mucosae, and it is placed under the category of pTis in the TNM staging as carcinoma in situ: invasion of lamina propria (6,12). However, WHO 2019 defines CRA with HGD as having high-grade cytology along with complex architectural structures, but the assessment of single-cell tumoral invasion of the lamina propria can be challenging. Some authors prefer to use intramucosal adenocarcinoma terminology only if there is a single-cell invasion of the lamina propria in CRAs (18). The data about these types of cases are scarce; in a study by Lewin et al., no metastatic disease was found in the follow-up or resection of 14 colorectal adenomas with lamina propria invasion and poorly differentiated components (19). The latest ICCR guidelines, published in 2020, state that in cases with lamina propria invasion the risk of metastasis is negligible even if they contain poorly differentiated components; it is recommended to classify these cases as HGD/non-invasive neoplasia, not as intramucosal adenocarcinoma (14,19–21). However, in a CRA case that showed infiltration into the lamina propria in the form of individual cells and signet ring cells, metastasis was detected in the follow-up (22). Regarding the presence of signet ring cells in CRAs, ICCR guidelines state that these cases should be specified in the pathology report as “unknown clinical behavior” since the clinical behavior of these cases is not clear (14). Coinciding with the publication process of this manuscript, the online (beta) version of the 6th edition of the WHO classification of Tumours of the Digestive System was released. While retaining the two-tiered dysplasia grading system for colonic adenomas, it also recognizes intramucosal adenocarcinoma as a very rare (<1%) but distinct entity separate from high-grade dysplasia. This diagnosis is defined by unequivocal invasion confined to the lamina propria, such as single-cell infiltration, tumor budding, or overtly poor differentiation (23). None of our HGD cases met these criteria for intramucosal adenocarcinoma, including the case presented in Question 4/Figure 6, which was most frequently diagnosed by respondents as intramucosal carcinoma or carcinoma in situ (~81%). Sometimes involvement of submucosal lymphoglandular complexes mimics invasive carcinoma and creates difficulties in diagnosing (24). In these kinds of rare cases, it is recommended by ICCR that pathologists should comment as such- if invasive carcinoma cannot be excluded (14).

According to the 8th edition of Criteria of the Union for International Cancer Control and American Joint Committee on Cancer, endoscopically removed CRAs with negative margins are considered as pTis (Stage 0), and lymph node dissection or surgical resection is not recommended because the probability of nodal involvement is very low (25,26).

A “high-risk” or “advanced” adenoma is the term used in screening programs to define the features of 1) tubulovillous or villous adenomas, 2) any adenoma with HGD, or 3) adenomas that are ≥10 mm in size. The patients that have high-risk adenomas have a shorter recommended surveillance time. European Society of Gastrointestinal Endoscopy guidelines recommends surveillance colonoscopy after three years for these patients rather than five years, which is eligible for the low-risk patients (5). Therefore, the correct diagnosis of HGD is essential not to overuse colonoscopy, which wastes financial resources and exposes patients to unnecessary risks.

The standardization of the grading of dysplasia in CRAs has become important in patient management, and it is essential to use a unified approach while reporting these lesions. The clinical impact of pathology reports should not be underestimated. This is the first study to investigate the current approach to grading dysplasia in CRAs among pathologists nationwide and the strengths of our study include the participant number being relatively high, with all being actively working pathologists from different sides of the country, and the fact that it puts forth a need in daily pathology practice. A limitation of the study might be the small number of cases; however, in order to achieve a higher participation rate and ensure the completion of the questionnaire, we aimed to keep the survey as concise and time-efficient as possible. To compensate for this limitation, we selected highly illustrative cases covering a range of dysplastic changes for each category, reflecting demonstrative, reliable, and clear examples of dysplasia grading that were derived from a guideline agreed upon by many expert pathologists.

In conclusion, it is seen that current dysplasia grading recommendations in CRAs are not fully implemented in practice among pathologists in Türkiye. In contrast to current guidelines for CRAs, 1) Intramucosal adenocarcinoma is still a commonly used terminology, 2) High-grade cytological features are over-relied upon, and 3) The insufficient extent of architectural abnormalities is overcalled. Therefore, we believe it is essential to develop strategies promoting the adoption of current guidelines in practice that will have a significant impact on patient health and cost-effectiveness in public health strategies. As agreed by the majority (Question 9), a national polyp guideline seems to be necessary for an attempt to standardize the reporting of CRAs in Türkiye.

## Conflict of Interest

The authors report there are no competing interests to declare.

## Funding

The authors have no relevant financial or non-financial interests to disclose and no funds, grants, or other support was received.
